# A conserved oxalyl-coenzyme A decarboxylase in oxalate catabolism

**DOI:** 10.1080/15592324.2022.2062555

**Published:** 2022-05-05

**Authors:** Ninghui Cheng, Vincent Paris, Xiaolan Rao, Xiaoqiang Wang, Paul A. Nakata

**Affiliations:** a USDA-ARS Children’s Nutrition Research Center, Department of Pediatrics, Baylor College of Medicine, Texas, United States; b Department of Biological Sciences, BioDiscovery Institute, University of North Texas, Texas, United States; c State Key Laboratory of Biocatalysis and Enzyme Engineering, School of Life Sciences, Hubei University, Wuhan, P. R. China

**Keywords:** Arabidopsis, catabolism, Coenzyme A, decarboxylase, oxalate

## Abstract

The ability to biosynthesize oxalic acid can provide beneficial functions to plants; however, uncontrolled or prolonged exposure to this strong organic acid results in multiple physiological problems. Such problems include a disruption of membrane integrity, mitochondrial function, metal chelation, and free radical formation. Recent work suggests that a CoA-dependent pathway of oxalate catabolism plays a critical role in regulating tissue oxalate concentrations in plants. Although this CoA-dependent pathway of oxalate catabolism is important, large gaps in our knowledge of the enzymes catalyzing each step remain. Evidence that an oxalyl-CoA decarboxylase (OXC) catalyzes the second step in this pathway, accelerating the conversion of oxalyl-CoA to formyl-CoA, has been reported. Induction studies revealed that OXC gene expression was upregulated in response to an exogenous oxalate supply. Phylogenetic analysis indicates that OXCs are conserved across plant species. Evolutionarily the plant OXCs can be separated into dicot and monocot classes. Multiple sequence alignments and molecular modeling suggest that OXCs have similar functionality with three conserved domains, the N-terminal PYR domain, the middle R domain, and the C-terminal PP domain. Further study of this CoA-dependent pathway of oxalate degradation would benefit efforts to develop new strategies to improve the nutrition quality of crops.

Oxalate is the simplest dicarboxylic acid and a common metabolite that is actively accumulated in some plants.^1^ In oxalate accumulating plants oxalate can be found as a soluble free acid and/or an insoluble metal oxalate crystal.^2^ It has been shown that oxalate can play important roles in various biological and metabolic processes such as detoxification of heavy metals, sequestration or balance of metal ions within the cell, and insect defense.

Although oxalate can have beneficial functions in the plant, oxalate present in edible plant tissues can have a negative impact on the animal or human consuming the plant food. In the soluble form oxalate can be absorbed directly from the diet. Humans and animals are unable to metabolize this acid, and thus, excrete it from the body to avoid physiological problems caused by this acid. It is during this process of excretion, however, that oxalate can complex with calcium, forming crystals, resulting in the pathological condition of renal/kidney stone formation.^3,4^ In the insoluble form, oxalate acts as an antinutrient binding calcium in the form of the calcium oxalate crystal. Calcium in this form is rendered unavailable for nutritional absorption by humans and animals^6–9^ thus lessening the nutritional value of several important plant foods. From a human health standpoint, reducing the consumption of oxalate would be desirable.^10^ A potential mechanism to reduce oxalate in plant foods is through its degradation.

There are three pathways of oxalate degradation that have been shown to occur in nature.^11^ These three pathways are catalyzed by oxalate decarboxylase, oxalate oxidase, and oxalyl-CoA synthetase. Oxalate decarboxylase activity has been extensively reported in fungi and some bacteria^11^ and a putative oxalate decarboxylase gene was recently reported in spinach,^12^ but validation via activity measurement remain to be conducted. Oxalate oxidase has been shown to breakdown oxalate into CO_2_ and H_2_O_2_.^13^ However, oxalate oxidase appears to be functional only in monocots. Taken together these findings suggested the possible existence of an alternative mechanism to degrade oxalate. Recent studies^14−19^ support a third CoA-dependent oxalate degradation pathway. This putative pathway has been proposed to consist of four enzymes, oxalyl-CoA synthetase (AAE3), oxalyl-CoA decarboxylase (OXC), formyl-CoA hydrolase (FCH), and formate dehydrogenase (FDH), that convert one molecule of oxalate into two molecules of CO_2_.^15^ Oxalyl-CoA synthetase (AAE3), the enzyme catalyzing the first step in this alternative pathway of oxalate catabolism, has been extensively studied in plants and other species.^16–18,20^

Recently, an oxalyl-CoA decarboxylase (OXC) was discovered in maize and Arabidopsis and shown to catalyze the second step in the CoA-dependent pathway of oxalate degradation.^14,19^ *OXC* was first identified in the prokaryotic gut bacterium *Oxalobacter formigenes* and shown to catalyze the conversion of oxalyl-CoA into formyl-CoA in a thiamine PPi-dependent decarboxylation manner.^21^ Coupling with formyl-coenzyme A transferase (FCR), OXC plays a critical role in oxalate degradation in bacteria.^22^ Within the human gut microbiota OXCs have been found to be highly conserved and can be separated into seven clusters.^23^

To analyze the evolutionary relationship of OXCs from various species, *OXC* protein sequences were identified in public databases and found to be widely spread and distributed in eukaryotes. The phylogenetic relationship and evolutionary rate of these OXC proteins were also highly conserved (Figure 1). Phylogenetic analysis clearly indicated that OXC proteins were clustered by organism classification (bacteria, fungi, plants and animals). Interestingly, the fungal (yeast) OXC was linked closer to animals than plants. In addition, OXC proteins showed an evolutionary separation in plants between dicots and monocots (Figure 1).

**Figure 1.**
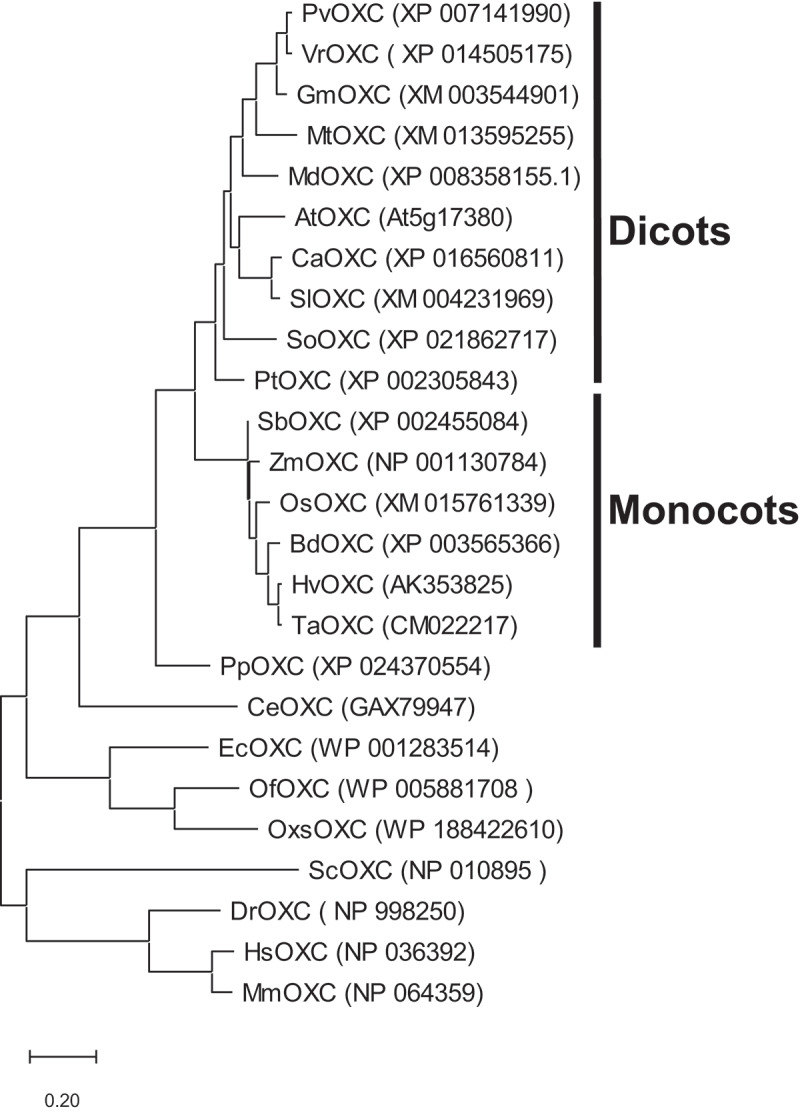
**Phylogenetic analysis of OXC proteins**. The OXC protein sequences were aligned using the ClustalW algorithm. The maximum likelihood phylogenetic tree was constructed using the MEGA 11.0 program. OXC proteins were derived from plant dicots: *Arabidopsis thaliana* (AtOXC), *Capsicum annuum* (CaOXC), *Solanum lycopersicum* (SlOXC), *Populus trichocarpa* (PtOXC), *Glycine max* (GmOXC), *Phaseolus vulgaris* (PvOXC), *Medicago truncatula* (MtOXC), *Spinacia oleracea* (SoOXC), *Vigna radiate* (VrOXC), *Malus domestica* (MdOXC); monocots: *Brachypodium distachyon* (BdOXC), *Oryza sativa* (OsOXC), *Hordeum vulgare* (HvOXC), *Sorghum bicolor* (SbOXC), *Zea mays* (ZmOXC), *Triticum aestivum* (TaOXC); Embrophyte: *Physcomitrium patens* (PpOXC); Chlorophyte: *Chlamydomonas reinhardtii* (CeOXC); Bacteria: *Escherichia coli* (EcOXC), *Oxalobacter formigenes* (OfOXC), *Oxalicibacterium solurbis* (OxsOXC); Fungi: *Saccharomyces cerevisiae* (ScOXC); Animals: *Danio rerio* (DrOXC), *Mus musculus* (MmOXC), *Homo sapiens* (HsOXC).

In agreement with the phylogenetic analysis, multiple OXC amino acids sequence alignments showed higher amino acid sequence identity among prokaryote (bacteria) and eukaryote than between these two groups (Figure 2). For example, the AtOXC shared an overall sequence identity of 40% and 43%, respectively, to the OfOXC and the EcOXC, and a 73% identity to the ZmOXC (Figure 2). Despite the difference in prokaryote and eukaryote sequence identity, molecular modeling suggests a conservation in overall OXC structure. (Figure 3). In each case the predicted model contains three conserved domains, including the N-terminal PYR domain, the middle R domain, and the C-terminal PP domain (Figure 3).

**Figure 2.**
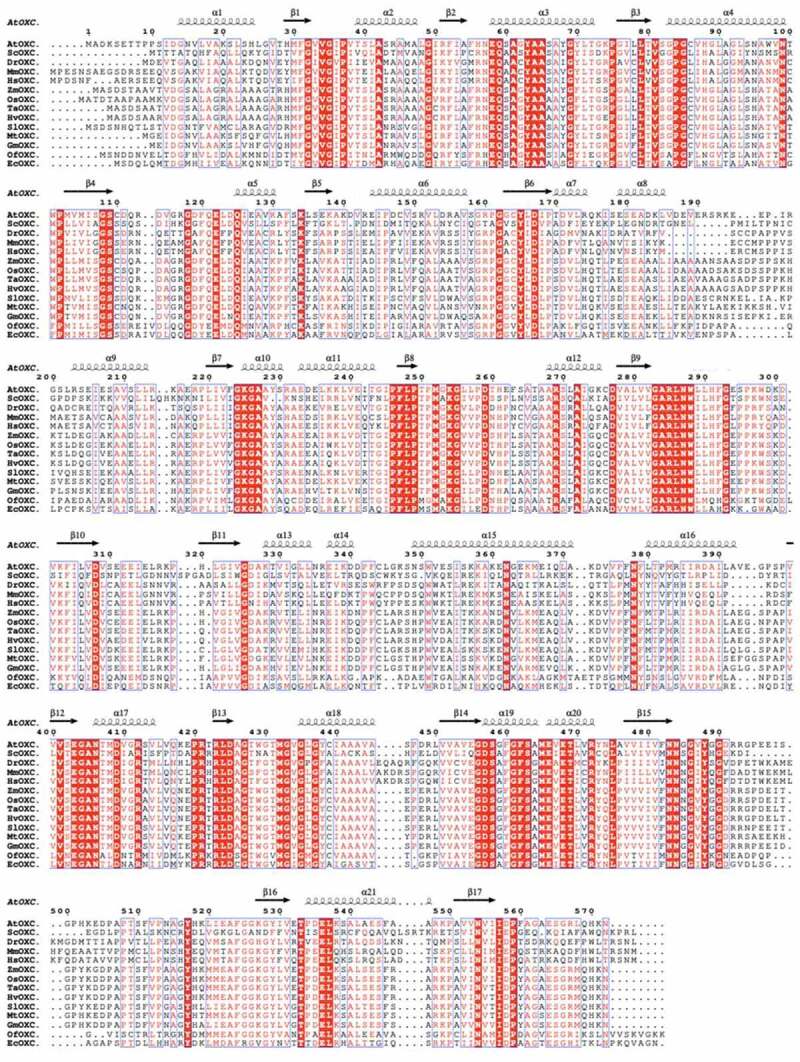
**Comparison of the predicted amino acid sequences of OXCs from bacteria, fungi, plants, and animals**. Sequence alignment of OXCs from *Arabidopsis thaliana, Saccharomyces cerevisiae, Danio rerio, Mus musculus, Homo sapiens, Zea mays, Oryza sativa, Triticum aestivum, Hordeum vulgare, Solanum lycopersicum, Medicago truncatula, Glycine max, Oxalobacter formigenes*, and *Escherichia coli* was performed using ClustalX^24^ and rendered by ESPript.^25^ The secondary structure elements observed in the AtOXC modeled structure are shown above the alignment. Conserved residues are highlighted.

**Figure 3.**
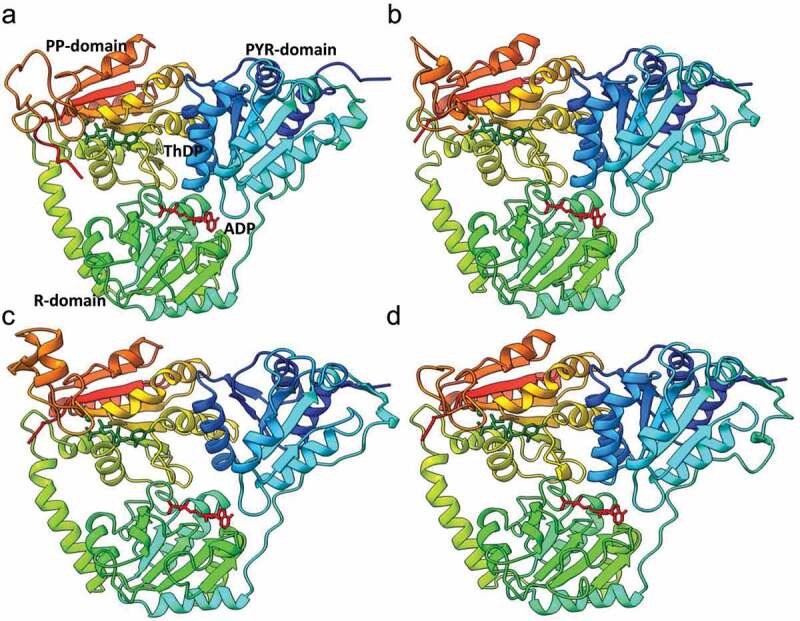
**Molecular modeling of OXCs**. Modeled structures of AtOXC (a), OsOXC (b), HsOXC (c), and ScOXC (d) docked with cofactors ThDP and Mg^2+^ ion, and activator ADP. Both ThDP (green) and ADP (red) are shown as stick models, and Mg^2+^ ion shown as a sphere model in green. The comparative models were generated using the Swiss-Model online server .^26^ and figures produced using ChimeraX.^27^

Expression of the gene encoding the AAE3 enzyme (oxalyl-CoA synthetase) was shown to be inducible by oxalate in Arabidopsis^15^ and *Medicago truncatula*.^16^ Likewise, expression of the maize^19^ and Arabidopsis^14^ OXCs were found to be induced by exogenous oxalate stimulation. Q-RT-PCR analysis of *OXC* expression in spinach, tomato, and rice also revealed an increase in transcript abundance upon exposure to an exogenous oxalate supply (Figure 4). Interestingly, our studies showed that both *AtOXC* and *SlOXC* were strongly induced by exogenous oxalate treatment, while the induction of *SoOXC* and *OsOXC* expression was relatively weak, but significant compared to controls. The reason for these differences in gene expression is currently unknown. One possible explanation is that there are simply differences in oxalate metabolism among the various plant species resulting in the measured differential response to exogenous oxalate supply. Such a difference may also explain the variability of oxalate concentrations in different plants, at least in part. Oxalate concentrations are generally higher in spinach and rice compared to Arabidopsis and tomato. Nevertheless, these findings suggest that regulation of *OXC* expression and most likely the other oxalate CoA-dependent catabolic pathway genes are functionally conserved in dicots and monocots.

**Figure 4.**
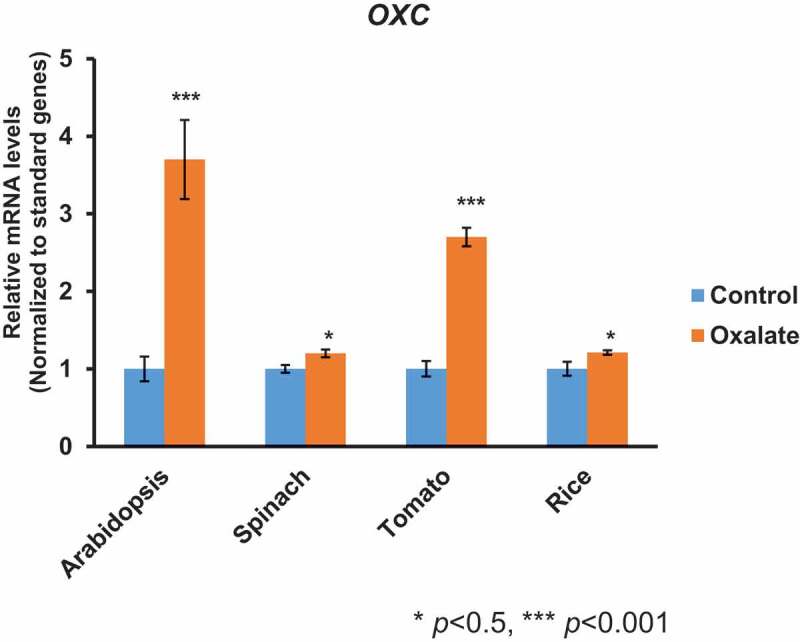
***OXC* expression in plants under oxalate stimulation**. qRT-PCR analysis of *AtOXC* (*UBQ10* was used as an internal control), *SoOXC* (Actin 1 was used as an internal control), *SlOXC* (*SlPP2Ac2*, encoding a protein phosphatase 2A catalytic subunit, was used as an internal control), and *OsOXC* (the basal transcription factor IIA gamma subunit 5 was used as an internal control) gene expression in 2-week-old seedlings. Student *t* test, n = 6, * p < .05, *** *p *< .001, indicating a significance between control vs oxalate treatment.

Structure-function analysis of OfOXC showed that Glu 62 (Glu 58 in AtOXC) is critical for proton transfer and enzymatic activity.^28^ A comparison of various OXC sequences revealed that Glu 62 is conserved across species supporting its universal importance (Figure 2). Three residues in the bacterial OfOXC, Tyr126, Glu127, and Tyr485, have been shown to reside in the putative active site of the enzyme and were predicted to be important for catalysis.^28,29^ Indeed, recent mutagenesis studies of the bacterial OXC supported this prediction.^23^ Interestingly, the three corresponding residues in eukaryotic OXCs (Figures 2 **and** Figure 3) such as the AtOXC, are Phe 120, Gln 121, and Tyr 488, respectively. The model predicts that these amino acids come from 2 OXC subunits suggesting that this enzyme functions as a dimer.^14^ The dimer composition is supported by molecular size estimates conducted using size exclusion chromatography.^14^ In this model, the ThDP cofactor resides between the PYR and PP domains of the two subunits, with its phosphate group interacting with Phe379 and Asn 406, its pyrimidine ring interacting with Glu58 and His88 of the other subunit, and the divalent Mg^2+^ ion cofactor interacting with Asp457 and Asn484.^14^ In addition, the activator, ADP, is predicted to sit in a cleft formed by the PYR, R, and PP domains where it interacts with the neighboring hydrophilic residues Arg160, Lys225, Arg285, Asp308, and Thr428.^14^ In contrast, a SAXS study conducted using the EcOXC suggests that the catalytically active prokaryotic enzyme assumes a tetrameric state.^30^ Future studies will help to elucidate the roles of specific amino acids in ligand binding, catalysis, and subunit multimerization.

Interestingly, human and mouse *OXC* homologs^31^ encode a peroxisomal enzyme, called 2-hydroxyacyl-CoA lyase (HACL1). HACL1 has been shown to be critical in the α-oxidation pathway, in which it can catalyzes 2-hydroxyacyl-CoA into formyl-CoA and pristanal in a thiamine pyrophosphate-dependent manner.^31^ Amino acid sequence alignment and molecular modeling analysis indicated HsOXC and MmOXc (HACL1) are similar to OfOXC, ScOXC, and plant OXCs (Figure 2 **and** Figure 3). Attempts to express HsOXC in *E. coli*, however, failed to yield an active enzyme.^31^ Thus, whether animal OXC homologs possess an oxalyl-CoA decarboxylase activity in *vivo* remains an open question for future study.

The conservation of OXC homologs in plants suggests that the CoA-dependent oxalate-degrading pathway is most likely also conserved in plants. Oxalate metabolism and its accumulation have been shown to be closely linked to both biotic and abiotic stress.^32^ Although direct evidence supporting a role for OXC in stress response is sparse, evidence is accumulating supporting a role for *AAE3*, which encodes an oxalyl-CoA synthetase that catalyzes the first step of this CoA-dependent pathway of oxalate catabolism, in pathogen resistance, metal tolerance, and other stress responses.^16,17,33^ Likewise, *FDH*, which encodes a formate dehydrogenase with an activity capable of catalyzing the last step in the proposed CoA-dependent pathway of oxalate catabolism, has been shown to be strongly induced under different types of plant stress, especially under conditions of hypoxia.^34,35^ It is conceivable that this oxalate-degrading pathway plays an important role in regulating plant stress responses through the modulation of oxalate catabolism.

Previous studies have shown that both AAE3 and OXC reside in the cytoplasm of the cell.^14,15,19^ Although a formyl-CoA hydrolase (FCH) catalyzing the third step in the CoA-dependent pathway of oxalate catabolism has been proposed, it has yet to be identified and characterized. The FDH proposed to catalyze the last step in the CoA-dependent pathway of oxalate catabolism has been shown to localize to the mitochondria.^34–36^ One possible explanation for differences in the compartmentation of these enzymes is that the CoA-dependent pathway does not proceed to the breakdown of oxalate to two molecules of CO_2,_ but rather to one molecule of CO_2_ and formyl-CoA where the latter is consumed in another biochemical pathway. Of course further investigations are required before any conclusion can be drawn.

## Materials and methods

### Reagents

All chemicals were purchased from Sigma-Aldrich (St. Louis, MO, USA) with the exception of Murashige and Skoog (MS) medium which was purchased from Caisson Laboratories Inc. (North Logan, UT, USA).

**Bioinfomatic analysis**The OXC protein sequences were aligned using the ClustalW algorithm. The maximum likelihood phylogenetic tree was constructed using the MEGA 11.0 program.^37^ Sequence alignment of OXCs were performed using ClustalX^24^ and rendered by ESPript.^25^ The OXC comparative modeling study was carried out using the Swiss-Model server^26^ and the figures prepared using ChimeraX.^27^

## Plant growth conditions, RNA isolation, cDNA synthesis, and qRT-PCR analysis

Arabidopsis wild type (ecotype Columbia, Col-0) seeds were surface-sterilized, germinated, and grown on one-half strength Murashige and Skoog (MS) medium (plus 0.5% sucrose) solidified with 0.8% agar for two weeks at 22°C under a 16 hr light and 8 hr night cycle. Seedlings were treated with 1/30 MS solution with or without addition of 1.5 mM sodium oxalate (pH 5.8) for 1 hr. For spinach, tomato, and rice, seeds were sown on commercial soil (Pro-Line, growing mix, C/20, Jolly Gardener, Oldcastle Lawn & Garden, Inc., ME, USA) in 16 cm pots. Two-week old seedlings were removed from the pots and then treated with 1/30 MS solution with or without sodium oxalate. Spinach and tomato plants were treated with 3 mM sodium oxalate (pH5.8) for 6 hr while rice plants were treated with 10 mM sodium oxalate (pH5.8) for 6 hr. Three independent experiments were conducted in which ten to twenty seedlings from each treatment were pooled for RNA isolation. Gene expression analysis was conducted following published procedures.^38,39^ In brief, total RNA was extracted using the QIAGEN RNeasy Plant Mini Kit. Five µg of the isolated total RNA was treated with DNase I. Two µg of the DNase I-treated RNA was then converted to cDNA using random hexamers and reverse transcriptase. The resulting cDNA was diluted to 250 ng/µL and 2 µL of cDNA used as a template for each qRT-PCR reaction. qRT-PCR was performed using the SYBR Green-based system and the Bio-Rad CFX96™. CFX Maestro Software for data collection and analysis. Relative mRNA levels were normalized to an internal reference. Statistical analysis was conducted using a two-way ANOVA. Primers used for each reaction are listed in Supplemental Table 1.

## Supplementary Material

Supplemental Material
